# Regulation of N6-methyladenosine (m6A) RNA methylation in microglia-mediated inflammation and ischemic stroke

**DOI:** 10.3389/fncel.2022.955222

**Published:** 2022-08-04

**Authors:** Fangfang Zhang, Yuanyuan Ran, Muhammad Tahir, Zihan Li, Jianan Wang, Xuechai Chen

**Affiliations:** ^1^Beijing International Science and Technology Cooperation Base for Antiviral Drugs, Faculty of Environment and Life, Beijing University of Technology, Beijing, China; ^2^Department of Rehabilitation, Beijing Rehabilitation Hospital, Capital Medical University, Beijing, China

**Keywords:** RNA methylation, ischemic stroke, microglia, neuroinflammation, polarization

## Abstract

N6-methyladenosine (m6A) is the most abundant post-transcription modification, widely occurring in eukaryotic mRNA and non-coding RNA. m6A modification is highly enriched in the mammalian brain and is associated with neurological diseases like Alzheimer’s disease (AD) and Parkinson’s disease (PD). Ischemic stroke (IS) was discovered to alter the cerebral m6A epi-transcriptome, which might have functional implications in post-stroke pathophysiology. Moreover, it is observed that m6A modification could regulate microglia’s pro-inflammatory and anti-inflammatory responses. Given the critical regulatory role of microglia in the inflammatory processes in the central nervous system (CNS), we speculate that m6A modification could modulate the post-stroke microglial inflammatory responses. This review summarizes the vital regulatory roles of m6A modification in microglia-mediated inflammation and IS. Stroke is associated with a high recurrence rate, understanding the relationship between m6A modification and stroke may help stroke rehabilitation and develop novel therapies in the future.

## Introduction

A stroke occurs when the blood supply to any portion of the brain stops, and it could happen due to blockage or injury to the blood vessels that carry blood to the brain. It is one of the leading causes of death and long-term impairment worldwide (Lo et al., 2003). Hemorrhagic and IS are the two main types of strokes. Hemorrhagic stroke is caused by a rupture of a blood vessel inside the brain, and ischemic stroke (IS) results due to the blockage of an artery in the brain, but both could lead to local hypoxia and damage to brain tissue (Barthels and Das, 2020). Among both types of strokes, IS account for roughly 80% of all stroke cases (Zheng et al., 2020). Various risk factors, including high blood pressure, obesity, and previous transient ischemic attack, could lead to an IS which could cause ischemic neuronal injuries such as glutamate excitotoxicity, oxidative stress, apoptosis, and neuroinflammation (Hankey, 2017; Dichgans et al., 2019).

Microglia/macrophages (MMs)-mediated neuroinflammatory responses could contribute to subsequent secondary damage following stroke (Liu et al., 2019b). MMs play a vital role in maintaining brain homeostasis, and these are the first line of defense against any damage to the central nervous system (CNS). In response to the variations in the cerebral microenvironment, microglia are activated and undergo morphological and functional changes. During the initial phase of IS, microglia are polarized to a protective anti-inflammatory phenotype and then gradually shifted to a toxic pro-inflammatory phenotype (Hu et al., 2012). It has been revealed that pro-inflammatory microglia trigger brain damage, hinder neurogenesis, and interfere with the recovery and repair of neurological function following stroke. On the contrary, the anti-inflammatory microglia could repair damaged tissues through various mechanisms, including neurogenesis, axonal remodeling, and remyelination (Bassett et al., 2021; Gaire, 2021). *In vivo and in vitro* studies showed that neuroprotection could be achieved by inhibiting the pro-inflammatory microglia and stimulating the activities of anti-inflammatory microglia (Liu et al., 2019b).

Many drugs have been reported to protect against brain injury by regulating microglia polarization and inflammatory responses in ischemia or hypoperfusion models (Liu et al., 2017, 2019b; Ran et al., 2021). Various physiological factors, such as hypothermia, could alter the microglia phenotype and provide neuroprotection in the state of stroke (Liu et al., 2018). Exploring the approaches that could shift microglia phenotype from pro-inflammatory to anti-inflammatory may provide the opportunity to develop novel therapeutic strategies for IS.

It is necessary to understand the occurrence and development of IS and determine its biological characteristics to improve its treatment efficiency. In recent years, it was reported that epigenetic modifications might dynamically regulate the changes in MM’s phenotype, which provided new clues for treating IS. Epigenetic modifications such as DNA methylation, histone acetylation, and non-coding RNA regulation have been identified for their close association with microglial polarization (Guo et al., 2016; Rigillo et al., 2018; Li et al., 2020). RNA methylation refers to the selective addition of methyl groups at different positions on RNA. N6-methyladenosine (m6A) and C5-methylcytidine (m5C) are the most common types of RNA methylation (Wang et al., 2015; Han et al., 2021). m6A RNA methylation occurs at nitrogen number six of the adenine base, and it is the most abundant chemical modification in eukaryotic messenger RNA (mRNA) and long-chain non-coding RNA (Filippova et al., 2019; Muller et al., 2019; van Tran et al., 2019; Ontiveros et al., 2020; Wen et al., 2020). It is known to modulate a variety of post-transcriptional modification processes such as RNA translation, splicing, and stability. Despite recent advances in m6A modification research, it is still worthwhile to discover new m6A sites and their functional capabilities. Reports showed that m6A modification plays a crucial role in the regulation of critical biological processes and pathogenesis of a variety of neurological diseases, such as Alzheimer’s disease (AD) (Han et al., 2020; Shafik et al., 2021), Parkinson’s disease (PD) (Chen et al., 2019; Qin et al., 2020; Qiu et al., 2020) and gliomas (Xu et al., 2020b; Zhang et al., 2020b). Recently, it has been reported that transient focal ischemia could alter the cerebral m6A epitranscriptome, which indicates the involvement of m6A modification in the onset of stroke (Chokkalla et al., 2019). Furthermore, studies have identified a distinct m6A epi-transcriptome in microglia. Microglia are critical regulators of inflammatory processes in the CNS. m6A modification could affect microglia-mediated inflammatory responses following stroke (Li et al., 2021).

This review summarizes the recent studies describing the role of m6A modification in IS and discusses the functions of m6A modification in microglia-mediated inflammation. Moreover, we also discuss the vital role of m6A modification in regulating the classical activation of microglia/macrophages (M1) and alternative activation of microglia/macrophages (M2). It has been noted that m6A modification could potentially play a role in inhibiting microglia-mediated inflammation following stroke. These findings may provide worthwhile insight into identifying m6A-related molecular biomarkers and therapeutic targets in stroke.

## Reversible/dynamic m6A RNA methylation in neurological diseases

### Reversible/dynamic regulation and physiological function of m6A RNA methylation

**FIGURE 1:**
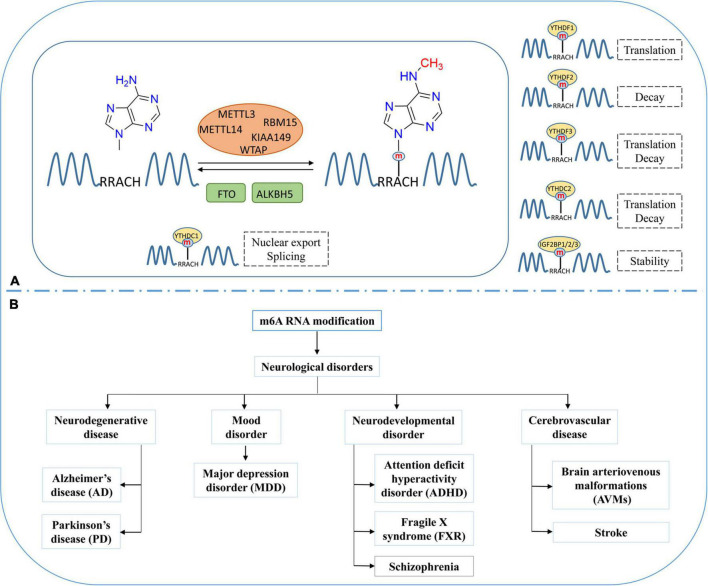
Dynamic and reversible regulation of m6A RNA modification and its role in neurological disorders. **(A)** Regulation of m6A modification by methyltransferases (METTL3, METTL14), demethylases (FTO and ALKBH5) and the effects of m6A modification on the processing, splicing, stability, and translation of mRNA. **(B)** Various neurological disorders regulated by m6A RNA modification.

m6A is the most prevalent modification in eukaryotic RNAs (Meyer and Jaffrey, 2017). On average, every transcript is estimated to contain two or three m6A-modified sites (Wang et al., 2020b). m6A RNA methylation is reversible, and dynamically regulated by methyltransferases (Writers) and demethylases (Erasers) (Figure 1A; Zhao et al., 2020). m6A methyltransferases are generally referred to as m6A methylase complex. It contains methyltransferase-like 3 (METTL3), methyltransferase-like 14 (METTL14), Wilm’s tumor-associated protein (WTAP), RNA-binding motif protein 15 (RBM15 and RBM15B), and KIAA1429 or VIRMA (vir-like m6A methyltransferase associated) (Zaccara et al., 2019; Zhao et al., 2020). S-adenosyl methionine (SAM) is a universal methyl-group donor in DNA, RNA, and histone methylation. METTL3 could combine with SAM and mediate RNA methylation in the nucleus. METTL14 acts as an RNA-binding platform and facilitates methyltransferase activity by forming a heterodimer with METTL3 (Zhang, 2018). WTAP is an essential adaptor protein that stabilizes the METTL3-METTL14 complex, and RBM15/15B and KIAA1429 help recruit the m6A methylase complex (He et al., 2019; Chen and Wong, 2020; Chen et al., 2020). At present, fat mass and obesity-associated protein (FTO) and AlkBHomolog5 (ALKBH5) are the only two demethylases that have been identified to remove the m6A modification of nuclear RNA (Deng et al., 2018; Wei et al., 2018; Zhang et al., 2020b).

m6A RNA methylation occurs in RRACH conserved sequences (R = A or G; and H = A, C, or U), frequently found near stop codons or 3′untranslated regions (3′UTRs). The function of m6A are regulated by m6A binding proteins (Readers), which can recognize and bind to the m6A modification sites at RNA and play an essential role in different biological functions (Lee et al., 2020). The YTH domain family, including YTH domain family protein 1–3 (YTHDF1/YTHDF2/YTHDF3) and YTH domain containing 1–2 (YTHDC1/YTHDC2), have m6A-binding domains that could bind to m6A modified RNA (Dai et al., 2021). YTHDF1 and YTHDF3 improve the translation efficiency of RNA by combining with translation initiation factor and ribosome (Liu et al., 2020; Shi et al., 2021). YTHDF2 is one of the most widely studied m6A binding proteins, which recognizes and combines with m6A modified mRNA to regulate its stability (Mapperley et al., 2021; Wang and Lu, 2021). Heterogeneous nuclear ribonucleoproteins (hnRNPs) are another kind of m6A binding protein. YTHDC1 and hnRNPs can recruit splicing factors to regulate pre-mRNA splicing (Martinez-Contreras et al., 2007; Kasowitz et al., 2018), whereas YTHDC2 accelerates the degradation of modified mRNA and enhances the translation of corresponding proteins via binding to targeted m6A modified RNA (Meyer and Jaffrey, 2017; Ma et al., 2019). Insulin-like growth factor-2 mRNA-binding proteins (IGF2BPs), including IGF2BP1/2/3, can identify m6A modification and promote mRNA stability and translation by protecting m6A-containing mRNA from degradation (Huang et al., 2018; Hu et al., 2020; Yang et al., 2020).

m6A methylation occurs after RNA transcription. In the nucleus, m6A modified RNA can be recognized by specific “Readers” that facilitate their splicing or nuclear export. After exporting to the cytoplasm, the cytoplasmic “reader” proteins could affect the translation, stability, and degradation of m6A modified mRNA (Jiang et al., 2021). Studies have shown that the m6A modification in RNA influences the regulation of many biological processes, including cell differentiation and proliferation, CNS and reproductive system development, and aging (Zhang et al., 2020a).

### m6A RNA methylation in neurological diseases

RNA methylation occurs in abundance in the mammalian brain and involves in the regulation of synaptic plasticity, learning, memory, and neurogenesis (Mathoux et al., 2021). The imbalance of m6A modification could develop various neurological disorders such as neurodegenerative disorders, mood disorders, neurodevelopmental disorders, and cerebrovascular disease (Figure 1B; Dermentzaki and Lotti, 2020). Neurodegenerative disorders like AD and PD are characterized by progressive neuronal loss in the brain. As reported in AD and related dementias, the FTO gene showed a greater risk than the other m6A-associated genes. Reitz et al. (2012) found a significantly lower level of FTO in cortex and amygdala tissue obtained from AD patients than in controls individuals. Recently, Han et al. (2020) observed increased m6A modification in the AD mice models. This finding indicates that m6A modification could play a vital role in developing AD (Han et al., 2020). Moreover, it is observed that the increased expression of METTL3 and the decreased expression of FTO could increase the m6A modification rate in AD models (Han et al., 2020). In YTHDF1-knockout mice, ectopic expression of YTHDF1 could alleviate AD-related symptoms, including learning defects and weak memory (Dermentzaki and Lotti, 2020). PD is another common neurodegenerative disease. Recent studies have revealed the potential relationship between m6A and the pathogenesis of PD (Rasheed et al., 2021). Anti-apoptotic effects following FTO knockdown have been observed in PD cell models (Chen et al., 2019). Moreover, increased FTO expression promoted m6A demethylation and induced apoptosis. These findings indicate that m6A modification and its regulatory proteins can control the survival of dopaminergic neurons and influence the incidence of PD (Selberg et al., 2021).

Major depression disorder (MDD) is one of the common mood disorders, and literature shows that ALKHB5 activity could increase the risk of MDD (Du et al., 2015). Neurodevelopmental disorders are a group of disabilities that appear during early development, such as attention deficit hyperactivity disorder (ADHD), fragile X syndrome (FXR), and schizophrenia. Studies have shown the association of m6A modification or m6A-related proteins with these neurodevelopmental disorders (Choudhry et al., 2013; Yoon et al., 2017; Edens et al., 2019). In cerebrovascular disease, brain arteriovenous malformations (AVMs) are congenital vascular abnormality. Wang et al. (2020a) found that the expression of METTL3 and WTAP decreased in the larger pathological tissues of AVMs. Stroke affects the entire brain and its network properties and is considered a network neurological disease. Recently, many studies have shown that stroke is regulated by RNA methylation. Therefore, we summarize the recent studies describing the role of the m6A modification in stroke and microglia-mediated inflammation.

## m6A RNA methylation in ischemic stroke

**FIGURE 2:**
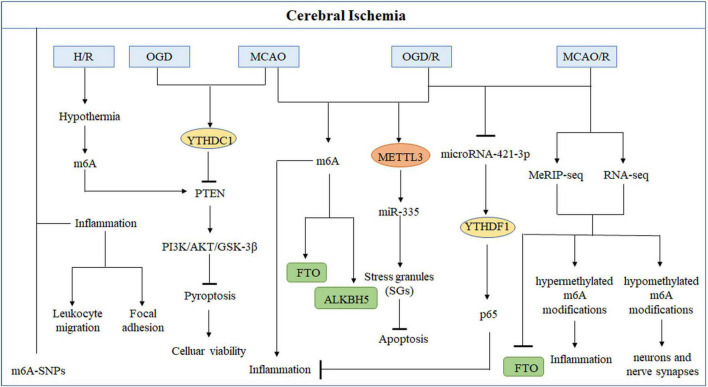
Role of m6A modifications in cerebral ischemia. Methyltransferases or demethylases regulated modulations in the levels of m6A modification and the effects of m6A binding proteins in the occurrence and development of stroke in different types of cerebral ischemia models.

Epigenetic modifications, including DNA methylation, histone modification, and non-coding RNAs, could play a role in the pathogenesis of IS. The role of m6A modification in ischemic brain damage remained unclear. Recently, several studies showed that m6A modification could regulate the expression of inflammation-related genes during the stroke (Figure 2 and Table 1). Analyzing the genes and regulatory mechanisms related to m6A modification in IS may be of great significance for an in-depth understanding of the pathogenesis and rehabilitation of stroke.

**TABLE 1:** The regulation of m6A RNA modification and its potential mechanism in ischemic stroke (IS).

m6A modulator	Model	IS-associated factors	Potential mechanisms	Association between m6A modification and IS	References
METTL3	MCAO, OGD/R	miR-335	Promotes SG formation	Negative	Si et al., 2020
FTO	MCAO/R	−	Inflammation, neurons and nerve synapses	−	Yi et al., 2021
FTO	MCAO, OGD/R	Bcl-2	Protect neurons apoptosis	Negative	Xu et al., 2020a
ALKBH5	MCAO, OGD/R	Bcl-2	Enhances neurons apoptosis	Positive	Xu et al., 2020a
YTHDF1	MCAO/R, OGD/R	P65	Prevent inflammatory response	Negative	Zheng et al., 2020
YTHDC1	MCAO, OGD	PTEN	Activated PI3K-AKT signaling	Positive	Zhang et al., 2020c
m6A	H/R-injured	PTEN	Activated the hippocampal neurons PI3K-AKT signaling	Positive	Diao et al., 2020
Global m6A modification	MCAO	−	Inflammation, apoptosis and transcriptional regulation	Positive	Chokkalla et al., 2019

### Changes of m6A-methyladenosine modification after ischemic stroke

It is observed that m6A modification increases in conditions like oxygen-glucose deprivation/reoxygenation (OGD/R) or middle cerebral artery occlusion and reperfusion (MCAO/R) (Chokkalla et al., 2019). Researchers found that the demethylases (ALKBH5 and FTO) primarily contribute to causing abnormal m6A modification in IS (Xu et al., 2020a; Yi et al., 2021). Yi et al. (2021) conducted a study in cerebral ischemia-reperfusion injury (CIRI) rat model to discover the variations in the m6A modifications and identified 1,160 differentially expressed genes having hypermethylated or hypomethylated m6A modifications. The genes that showed differential expression due to hypermethylation were found to participate in inflammation-related processes, while genes with hypomethylation modification were found to be associated with the functioning of neurons and nerve synapses. FTO, a demethylase, was explicitly present in neurons. Its downregulation was observed following MCAO/R, which caused elevation in the m6A level (Yi et al., 2021). Similarly, Xu et al. (2020a) found that in rat models, m6A levels increased with ALKBH5 expression in the cerebral cortex and primary neurons following MCAO and OGD/R, respectively. Moreover, FTO mRNA and protein levels were observed to be decreased in these rat models, which indicates that ALKBH5 and FTO work together to regulate m6A modification levels. ALKBH5 and FTO could selectively cause demethylation of Bcl-2 transcript; as a result, Bcl-2 expression is increased, which contributes to reduce neuronal damage. In the early stage of stroke, ischemia or exposure of neurons to hypoxia can temporarily halt the translation process, thereby reducing the development of cerebral ischemia and preventing additional damage. The translation arrest of proteins is essential for producing stress granules (SGs), which can immediately and temporarily halt mRNA translation. At an early stage of acute ischemic stroke (AIS), SGs will shield necessary mRNA and proteins from the potentially damaging environment, increasing cells’ survival (Aramburu-Nunez et al., 2022). Si et al. (2020) described that METTL3 promotes the maturation of miR-335, enhancing SG formation and reducing neuronal apoptosis. It showed that METTL3-mediated m6A methylation could alleviate AIS.

In these investigations, the m6A modification regulated by demethylases or methyltransferases was shown to play an essential role in IS. The incidence of stroke could increase intracellular m6A RNA methylation levels, which further regulates the expression of the stroke-related proteins and influences the activities of the corresponding signaling pathways. Therefore, m6A RNA methylation may bring new insights or prospective therapeutic options for IS in the future.

### Changes of m6A modification- related inflammation after cerebral apoplexy

In IS, a lack of oxygen and energy might set off a chain reaction of physiological and pathological processes, such as an inflammatory response, glutamate excitotoxicity, oxidative stress, and apoptosis, which would ultimately lead to severe brain damage (Zhao et al., 2017). Following the stroke, inflammatory reactions start due to reduced blood flow, activation of intravascular immune cells, and the production of pro-inflammatory molecules by the damaged endothelium and brain parenchyma. Inflammation could contribute to worsening the ischemia-related damage. Blood vessel endothelium and platelets experience stress due to reduced blood flow, leading to intravascular congestion and ischemia (De Meyer et al., 2016). Clogging contributes to the coagulation cascades’ activation, which further generates inflammatory cues (Lambertsen et al., 2019). Coagulation cascades lead to intravascular inflammation, monocytes, and complement system activation through a series of downstream events. Intravascular inflammation could disrupt blood-brain barrier (BBB) (Szeplaki et al., 2009; Alawieh et al., 2015). A damaged BBB could allow the passage of activated complement proteins and immune cells to the brain parenchyma. As a result, inflammatory reactions occur in the brain parenchyma, which causes damage to neurons. Injured and dying neurons produce danger-/damage-associated molecular patterns (DAMP) (Garcia-Bonilla and Iadecola, 2012; Benakis et al., 2014; Petrovic-Djergovic et al., 2016; Singh et al., 2016).

Within hours of initiating ischemia, microglia respond to the changing environment (Zhang et al., 2011). Activated microglia penetrate the peri-infarcted and infarcted sites. Resident and invading cells regulate the inflammatory responses following stroke by interacting with one another and damaged neurons via signaling molecules, such as cytokines. At various phases following IS, inflammatory responses can be harmful and beneficial. Although it can contribute to increasing infarcts, it is also crucial for infarct clearance and influences remodeling and healing. Several preclinical and clinical investigations have demonstrated the efficacy of pharmaceutical therapies that target inflammation following IS. Targeting specific inflammatory molecules, such as interleukins (IL-1, IL-6, IL-10) and tumor necrosis factors, has shown significant therapeutic potential in preclinical settings (Lambertsen et al., 2019).

Neuroinflammation can eliminate cell injury and remove debris in the process of cerebral ischemia, and initiate tissue repairment. Therefore, it can be used as a therapeutic target for cerebral ischemia (Skaper et al., 2018). More and more studies have shown that different inflammation-related cells play an important role in different stages of cerebral ischemia. As inflammatory response occurs accompanied with cerebral ischemia, neuroinflammation may lead to a secondary neuronal damage, which will aggravate ischemia injury and impede the chronic recovery of brain function (Jurcau and Simion, 2021).

Despite recent advances in stroke research there is only a little research on m6A modification in pathological conditions, including IS and vascular inflammation, but what is known so far shows that it plays a crucial role in stroke and warrants more investigation. Zhu et al. (2021) examined the link between m6A single-nucleotide polymorphisms (m6A-SNPs) and the risk of IS using integrative analysis of genome-wide association studies (GWAS) and a list of single-nucleotide polymorphisms (SNPs) from the m6AVar database. More than 80 local genes (containing 87 m6A-SNPs) were discovered to be differentially expressed in IS patients in many biological processes, such as leukocyte migration and focal adhesion. Because m6A-SNPs are always adjacent to the methylation site, changes in m6A modification may impact the underlying biological process (Zheng et al., 2018). The earliest indicators of vascular inflammation include leukocyte migration and focal adhesion. The enrichment analysis revealed that m6A modification might have a role in the progression of IS by controlling vascular inflammation.

In recent years, neuronal inflammatory response and pyroptosis have been implicated in the etiology of CIRI and may provide a therapeutic target in the future. Chokkalla et al. (2019) established a transient ischemia-reperfusion (I/R) mouse model to investigate the transcriptome-wide m6A modification in CIRI. They found that mRNAs with various m6A modification were shown to be more prevalent in biological processes such as inflammation, apoptosis, and transcriptional regulation. Furthermore, the key inflammatory pathways that exhibited enhanced m6A RNA modification were cytokine, tumor necrosis factor (TNF), Toll-like receptor (TLR), and nuclear factor-kappa B (NF-κB) signaling. These are all known to affect the inflammation that occurs after a stroke. Diao et al. (2020) demonstrated that hypothermia can protect neurons from CIRI-induced proptosis via m6A-mediated activation of phosphatase and tensin homologous protein (PTEN). Furthermore, hypothermia can reduce inflammatory damage by lowering the expression levels of the NLRP3 (NLR pyrin domain containing 3) inflammasome and its related cytokines. In addition, the critical genes that were downregulated were mostly implicated in the cAMP signaling pathway, neuroactive ligand-receptor interaction, and the dopaminergic synapse (Yi et al., 2021). These findings indicate that the m6A modification could reduce the damage to neurons and synapses following CIRI, which provides new insight into the strategies for safeguarding neurons after CIRI. High-throughput sequencing and low-throughput investigations demonstrated that stroke can alter the levels of m6A in the brain and that demethylases FTO and ALKBH5 are involved in regulation, in addition to alterations in m6A recognition proteins, such as the YTH family. Zheng et al. (2020) found that microRNA-421-3p prevents inflammatory response in CIRI by targeting YTHDF1 to inhibit p65 mRNA translation. Moreover, Zhang et al. (2020c) confirmed that knockdown of YTHDC1 could exacerbate ischemic brain injury. Mechanistically, YTHDC1 facilitated neuronal survival following ischemia by promoting the degradation of PTEN mRNA, which led to an increase in Akt phosphorylation. In addition, it was found that m6A modifications in inflammation-related genes altered in stroke, which led to the activation of inflammation-related pathways (Zhang et al., 2020c).

In a nutshell, elucidating the function of m6A modification in the inflammatory response may yield valuable evidence for molecular targeting research, which may improve the treatment of inflammation-mediated cerebral apoplexy.

## Role of microglia-mediated inflammation in ischemic stroke

Inflammation is one of the primary causes of IS, induced by developing reactive oxygen species (ROS), necrotic cells, or damaged tissues following cerebral ischemia (Khoshnam et al., 2017). Inflammation could cause neuronal apoptosis and brain injury in the acute phase of an IS through excitotoxicity, cytolysis, oxidative stress, and thrombotic inflammation (Shi et al., 2019). In IS models, microglia activation varies and is proportional to the severity of ischemia at various phases (Yu et al., 2021). Similarly, clinical trials demonstrated that microglial activation could occur in the acute, subacute, and chronic stages of IS (Qin et al., 2019). Numerous studies have shown that neuroinflammation caused by cerebral ischemia can influence the progression of cerebral ischemia in its later stages (Jayaraj et al., 2019). Therefore, neuroinflammation could be one of the therapeutic targets of cerebral ischemia that develops in the brain following ischemia.

MMs are the primary immune cells defending against CNS damage and are crucial in regulating brain homeostasis (Liu et al., 2019b; Qiu et al., 2021). After cerebral ischemia, local microglia and peripheral macrophages are quickly activated and regulate neuropathological processes to carry out functional recovery (Zhao et al., 2017; Li and Barres, 2018). Activated MMs play an important role in the pathological process of ischemic brain injury. To accomplish their diverse roles, MMs exhibit remarkable plasticity and can exhibit distinct active phenotypes depending on the microenvironment. There are two main phenotypes of activated MMs: pro-inflammatory (M1) and anti-inflammatory (M2) phenotypes. M1-like MMs have been shown to breach the BBB and aggravate neurological impairments by producing inflammatory cytokines like TNF, IL-1β, and inducible nitric oxide synthase (iNOS). In contrast, M2-like MMs support tissue repair by clearing cell debris, promoting neurogenesis, angiogenesis, axon regeneration, and releasing anti-inflammatory cytokines such as arginase-1 (Arg-1) and IL-10, and neurotrophic factors (Prinz et al., 2021; Qiu et al., 2021). Therefore, most of the current studies focus on the ways to inhibit M1 and/or enhance M2 phenotype to control the activation of microglia, to limit inflammatory injury and promote tissue repair to treat IS and improve neuronal survival.

## m6A RNA methylation in microglia/macrophage-mediated inflammation

Neuroinflammation is the inflammatory response occurs in CNS, and is closely related to the occurrence of neurological diseases (Hickman et al., 2018). In the pathological process of AD, the level of inflammatory markers were increased and aggravates the brain damage, which indicated that neuroinflammation has a prominent role in the pathogenesis of AD (Leng and Edison, 2021). Suppression of microglial activity in AD mice models improved neurogenesis and enhanced cognitive functions (Biscaro et al., 2012). Moreover, Growing evidence indicates that microglia-mediated inflammation also participates in the process of PD (Zhang et al., 2017; Saha et al., 2021). The findings suggest that microglia activation-mediated modulations in immune responses could play a significant role in progression of neurodegenerative disorders. During stroke occurrence and development, cerebral inflammation plays important roles and was seem as a breakthrough in treatment of stroke (Lyu et al., 2021). Recently, it is showed that epigenetics could regulate the inflammatory response, which suggests novel understanding in inflammation research (Qiu et al., 2021). To better understand the role of m6A modification in the inflammatory response in the brain, we then focus on the microglia/macrophages that generate an inflammatory response in the brain to gain new insights into neuroprotection following cerebral ischemia.

### m6A regulates the inflammatory response of microglia

Microglia act as vital communicators between CNS and immune systems. Li et al. (2021) created primary rat microglia models differentiated into M1, M2, and M0 (resting and unstimulated)-like phenotypes (M1-L, M2-L, M0-L) and compared their m6A modification profile of mRNA and lncRNA to demonstrate the regulatory role of m6A modification in microglia polarization. The findings revealed that 87 m6A-associated lncRNAs were hyper-methylated in M1-L/M0-L and hypo-methylated in M2-L/M1-L, with just three hypo-methylated lncRNAs in M1-L/M0-L and hyper-methylated in M2-L/M1-L. Moreover, pro-inflammatory M1-L exhibited the most remarkable shift in m6A RNA modification. KEGG analysis showed that nine overly expressed mRNAs with hyper-methylation in M1-L/M0-L were involved in 10 significantly upregulated pathways, such as signal transduction, immune system processing, and protein degradation. These pathways may represent diverse pro-inflammatory processes that can occur during microglial activation. These findings suggest that the lncRNA and mRNA with variable methylation can regulate the inflammatory responses in microglia by modulating immune system functioning and signal transduction pathways.

Inflammation, a vital component of the immune response, helps the body recover from damage or infection and maintains tissue homeostasis in potentially dangerous circumstances. On the other hand, if it continues uncontrollably, it could cause more tissue damage, chronic inflammatory, and autoimmune illnesses, and ultimately organ loss. Studies have demonstrated that microglia are the predominant cells responsible for the inflammatory response in the brain in the majority of neurodegenerative disorders (Smith et al., 2012; Tang and Le, 2016). Lipopolysaccharide (LPS), an early inflammation trigger, can be identified explicitly by Toll-like receptor 4 (TLR4) to activate intracellular signal transduction. Hence immunological and inflammatory responses are triggered. It has been reported that following LPS exposure, microglia activate and subsequently produce a large number of inflammatory agents and other cytotoxic chemicals, which leads to the death of dopaminergic neurons and significant damage to CNS (Zhou and Spittau, 2018). In microglial inflammation induced by LPS, the expression of METTL3 and the inflammatory cytokines were observed to be elevated (Ran et al., 2021; Wen et al., 2022). The tumor necrosis factor receptor-associated factor (TRAF) is a crucial binding protein in the TNF and Toll/IL-1 receptor (TIR) families. TRAF6, as a signal transduction factor, is also vital in the initiation of numerous signal cascades. NF-κB is a well-known pro-inflammatory signaling pathway and is considered an essential component in the regulation of both immunity and inflammation. Recent studies have stated that the TRAF6-NF-κB signaling mechanism significantly affects a variety of inflammatory responses (Allen et al., 2011). Wen et al. (2022) discovered that the level of METTL3 had a positive correlation with TRAF6, and METTL3 overexpression had the potential to activate the TRAF6-NF-kB pathway in an m6A-dependent manner, which would ultimately lead to microglial inflammation. Therefore, m6A modification could be a significant regulator during the immunological response of microglia.

### m6A participates in macrophage polarization and inflammation

**FIGURE 3:**
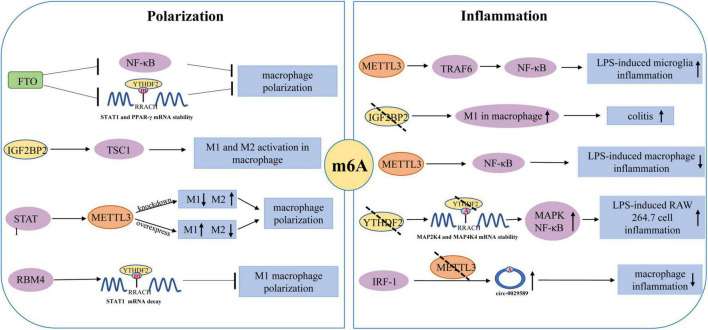
Regulatory role of m6A modification in the polarization of macrophages and macrophages/microglia mediated inflammation.

The polarization of macrophages is the driving force behind various inflammatory disorders, particularly those associated with an M1/M2 imbalance (Atri et al., 2018; Figure 3). Several transcription factors have been identified as essential regulators of macrophage polarization, including hypoxia-inducible factor-1 alpha (HIF-1α) and peroxisome proliferator activator receptor-γ (PPAR-γ), signal transducer and activator of transcription 1 (STAT1) and STAT6 (Funes et al., 2018). There is evidence that interferon-gamma (IFN-γ) could activate Jak-STAT1 signaling and promotes STAT1 phosphorylation, which leads to the polarization of M1-like macrophages. STAT6 signaling could trigger the expression of ligand-dependent PPAR-γ and promotes M2 polarization by promoting the expression of several gene subsets associated with anti-inflammatory function. The polarization of M1/M2 macrophages is driven by m6A modification. Due to a significant regulator of macrophage polarization, m6A modification may be utilized as an anti-inflammatory target. Gu et al. (2020) found that FTO silencing significantly inhibited the polarization of M1 and M2. FTO knockdown was observed to cause a significant decrease in the expression of STAT1 in M1 polarized macrophages and the expression of STAT6 and PPAR-γ in M2 polarized macrophages. Similarly, FTO knockdown caused the inhibition of the NF-κB signaling pathway and reduced the stability of STAT1 mRNA and PPAR-γ mRNA by activating YTHDF2, thus hindering the activation of macrophages (Gu et al., 2020). Liu et al. (2019a) found that METTL3 could cause M1 macrophage polarization through direct methylation of STAT1 mRNA. METTL3 knockdown significantly inhibited M1 polarization but enhanced M2 polarization. In addition, METTL3-mediated STAT1 mRNA methylation significantly increased the stability of mRNA and subsequently upregulated the expression of STAT1. Wang et al. (2021) observed that the polarization of M1 was enhanced by the IGF2BP2 knockdown in macrophages. Meanwhile, IGF2BP2 tended to transform the M1 phenotype into the M2 phenotype in an m6A-dependent manner. Moreover, it was also discovered that the IL-4-Jak-STAT6 axis could carry out the differentiation of M2 macrophages. Huangfu et al. (2020) found that RNA-binding motif 4 (RBM4) could interact with YTHDF2 to degrade STAT1 mRNA, thereby inhibiting the polarization of M1 macrophages. These findings prove that m6A modification plays a significant role in the polarization of macrophages.

Moreover, an *in vitro* study showed that LPS could promote the expression and biological activity of METTL3 in macrophages (Wang et al., 2019b). However, the overexpression of METTL3 can attenuate LPS-induced inflammation through the NF-κB signaling pathway. Yu et al. (2019) reported that YTHDF2 knockdown could increase the expression of MAP2K4, which results in the activation of MAPK and NF-κB signaling pathways. This activation leads to the enhanced expression of pro-inflammatory cytokines and aggravates the inflammatory responses of the cells. Pyroptosis, a kind of programmed cell death, occurs due to the release of many pro-inflammatory factors in response to the inflammatory reactions (Robinson et al., 2019). Guo et al. (2020) demonstrated that m6A modification in circRNA (hsa_circ_0029589) could enable IFN Regulatory Factor-1 (IRF-1) to induce macrophage pyroptosis in patients with the acute coronary syndrome (ACS). High levels of m6A modification in circRNA (hsa_circ_0029589) caused a decrease in circRNA (hsa_circ_0029589) expression and an increase in METTL3 expression in macrophages of ACS patients. On the contrary, METTL3 inhibition significantly increased the expression of circRNA (hsa_circ_0029589) and alleviated the pyroptosis of macrophages. However, additional research is required to investigate the underlying processes of m6A modification in macrophage pyroptosis-mediated inflammation.

## Therapeutic potential

m6A RNA modification is the most abundant type of epigenetic change in eukaryotic RNA. The function of m6A modification and its regulation in human disorders have undergone significant development over the past decade. It is notably true in neurological diseases caused by the enrichment of m6A in mammalian brains. Chokkalla et al. (2019) demonstrated for the first time that the global m6A levels in the whole cerebral transcriptome were altered after stroke. Many studies have reported that IS could occur due to imbalanced m6A RNA modification (Table 1). Recently, several studies exhibited that IS models have high levels of increase in m6A methylation, and m6A is found to reduce stroke-related damage. The regulation of m6A “Writers,” “Erasers,” and “Readers” may slow the progression of IS, and these findings may offer new insights into potential biomarkers or treatment options for stroke diagnosis and management. Many studies indicated that the up-regulated m6A level in IS is closely associated with the decreased expression of the demethylase FTO (Yi et al., 2021). Therefore, modulations in m6A levels and expression of FTO may be considered a bio-marker of IS development. Different studies reported the dual relationship, positive or negative, between m6A and IS, which may be due to the utilization of dissimilar IS models or targeting different m6A modified RNA. The level of m6A can affect the survival of neurons in the OGD/R model. Knockout of ALKBH5 could promote neuronal apoptosis, while the overexpression of FTO could promote the survival of neurons, indicating the neuroprotective role of FTO. At present, m6A modification regulatory drugs may be utilized for IS management. Recently it has been reported that cycloleucine (CL) is one kind of m6A inhibitor that can reduce nerve necrosis by inhibiting m6A modification (Xu et al., 2020a). Moreover, as a methyl donor, betaine can significantly increase the global m6A level and enhance the I/R brain injury by increasing m6A modification. Given the IS-regulatory role of m6A, using m6A inhibitory drugs may aid in managing IS.

The full transcriptome map of m6A in mRNA may help classify m6A targets and help understand the potential epigenetic mechanisms. Some studies have explained the effect of m6A-SNP on IS and identified the biological processes related to the pathogenesis of IS, such as inflammatory response. MeRIP-seq in the stroke model is also an important method for studying the pathogenesis of IS. Zhu et al. (2021) discovered that the primary biological processes that regulate post-stroke m6A differential methylation are inflammation, apoptosis, and transcriptional regulation. Other studies identified that m6A-influenced differential gene expression following stroke could affect inflammation, neuronal activity, and synaptic processes. In our study, we primarily focused on the regulatory role of m6A modification in the inflammatory process and its effects on inhibition or occurrence and progression of IS. The m6A-SNPs or MeRIP-seq mediated identification of the regulatory role of m6A-associated genes or signaling pathways in IS may help find potential biomarkers and therapeutic targets for IS.

Neuroinflammation is a complex pathophysiological process that includes microglial activation and the release of inflammatory cytokines. It is a significant contributor to neuronal damage and the pathogenesis of IS. Recent studies have shown that m6A modifications can promote neuroinflammation after a stroke and reduce the severity of the harm by controlling the inflammatory response. m6A modification plays a significant role in several inflammatory diseases via regulating various pathways, but the understanding of the inflammatory response in the disorders of the nervous system is insufficient. One of the most significant advances in treating stroke injury is identifying a secondary inflammatory reaction that occurs after a stroke. The investigation of m6A modification in inflammatory response could enable the development of effective drugs with particular targets. Curcumin is a yellow polyphenolic pigment extracted from turmeric. It is reported that curcumin has a protective effect on LPS-induced liver injury and lipid metabolism disorder (Lu et al., 2018). Dietary curcumin could control the mRNA expression of METTL3, METTL14, ALKBH5, FTO, and YTHDF2. It can also enhance the level of m6A modification and protect the liver from damage caused by inflammation and metabolic disorders. In addition, the natural product Rhein has been identified as an inhibitor of FTO. It could competitively bind to FTO active sites. It has been found that Rhein inhibited the ATP-induced inflammation in fibroblast-like synoviocytes in rheumatoid rat models and improved experimental colitis (Hu et al., 2019). Rhein treatment was also discovered to inhibit inflammatory lung injury caused by the human respiratory syncytial virus. A literature review showed that few studies investigated the regulatory function of m6A methylation in neuro-inflammation and its potential role in stroke treatment. In this review, we discussed some critical m6A regulatory factors which can change the phenotype and function of microglia/macrophages. There is still a need to conduct in-depth investigations of the regulatory role of m6A methylation in post-stroke neuro-inflammation. It could help us devise m6A modification-related novel therapeutic approaches to alleviate post-stroke inflammatory responses mediated damages.

In addition, aberrant m6A modification is involved in additional ischemia cascade activities, such as oxidative stress and apoptosis. In cerebral ischemia, plenty of reactive ROS are produced and cause oxidative stress and tissue damage. A study reported that METTL3-mediated m6A modification positively affects miR-873-5p, which regulates the Keap1-Nrf2 pathway and reduces oxidative stress (Wang et al., 2019a). It suggests that METTL3 may protect neurons from oxidative stress-induced damage, and it can be utilized as a treatment option to overcome oxidative stress produced by stroke. Excess glutamate synthesis after stroke promotes Ca^2+^ influx, which could cause excitotoxicity and neuronal damage. However, the role of m6A modification in glutamate-mediated excitotoxicity is not yet fully elucidated and requires further investigation.

m6A modification and its precise regulation in stroke remain mostly unclear and requires additional studies. Current investigations suggest that m6A modification may enable the identification of prospective targets for the treatment of stroke, leading to the development of promising m6A inhibitors or agonists for clinical use in the future. Furthermore, inhibition of post-stroke inflammation by targeting m6A methylation could help improve stroke-related injuries. We believe that m6A methylation may provide new insights into the molecular mechanism of IS and may serve as a potential therapeutic target for IS therapy.

## Conclusion

The dynamic and reversible m6A RNA methylation proved to have a significant regulatory role in crucial biological processes in eukaryotes. Recent studies have revealed that the dysregulated m6A methylation process or abnormal m6A levels are closely related to the pathogenesis of IS. Moreover, m6A modification can facilitate the activation and polarization of microglia/macrophages and could also play a regulatory role in microglia-induced inflammatory responses following stroke. The interaction between m6A methylation and IS could be helpful in a better understanding of the pathogenesis of IS and discovering novel therapeutic targets for IS treatment.

## Author contributions

XC raised the idea for the article, performed literature search, and revised the manuscript. FZ, YR, and MT wrote the first draft of the manuscript. ZL and JW revised the work. All authors contributed to the article and approved the submitted version.
